# *Lactobacillus crispatus* inhibits growth of *Gardnerella vaginalis* and *Neisseria gonorrhoeae* on a porcine vaginal mucosa model

**DOI:** 10.1186/s12866-015-0608-0

**Published:** 2015-12-09

**Authors:** Laura M. Breshears, Vonetta L. Edwards, Jacques Ravel, Marnie L. Peterson

**Affiliations:** Department of Experimental and Clinical Pharmacology, College of Pharmacy, University of Minnesota, 4-442 McGuire Translational Research Facility, 2001 6th St. SE, Minneapolis, MN 55455 USA; Institute for Genome Sciences, University of Maryland, School of Medicine, Bio Park II, 6th Floor, 801 West Baltimore St., Baltimore, MD 21201 USA

**Keywords:** *Lactobacillus*, Vaginal explants, Bacterial vaginosis, Microbiota, Sexually transmitted infections, Vaginal mucosa, Biofilm

## Abstract

**Background:**

The vaginal microbiota can impact the susceptibility of women to bacterial vaginosis (BV) and sexually transmitted infections (STIs). BV is characterized by depletion of *Lactobacillus* spp., an overgrowth of anaerobes (often dominated by *Gardnerella vaginalis*) and a pH > 4.5. BV is associated with an increased risk of acquiring STIs such as chlamydia and gonorrhea. While these associations have been identified, the molecular mechanism(s) driving the risk of infections are unknown. An *ex vivo* porcine vaginal mucosal model (PVM) was developed to explore the mechanistic role of *Lactobacillus* spp. in affecting colonization by *G. vaginalis* and *Neisseria gonorrhoeae*.

**Results:**

The data presented here demonstrate that all organisms tested can colonize and grow on PVM to clinically relevant densities. Additionally, *G. vaginalis* and *N. gonorrhoeae* form biofilms on PVM. It was observed that lactic acid, acetic acid, and hydrochloric acid inhibit the growth of *G. vaginalis* on PVM in a pH-dependent manner. *N. gonorrhoeae* grows best in the presence of lactic acid at pH 5.5, but did not grow well at this pH in the presence of acetic acid. Finally, a clinical *Lactobacillus crispatus* isolate (24-9-7) produces lactic acid and inhibits growth of both *G. vaginalis* and *N. gonorrhoeae* on PVM.

**Conclusions:**

These data reveal differences in the effects of pH, various acids and *L. crispatus* on the growth of *G. vaginalis* and *N. gonorrhoeae* on a live vaginal mucosal surface. The PVM is a useful model for studying the interactions of commensal vaginal microbes with pathogens and the mechanisms of biofilm formation on the vaginal mucosa.

## Background

Sexually transmitted infections (STIs) are a worldwide public health problem accounting for over 1 million newly acquired infections every day (World Health Organization). The two most commonly reported bacterial STIs in the United States are chlamydia (caused by *Chlamydia trachomatis)* and gonorrhea (caused by *Neisseria gonorrhoeae*) (Centers for Disease Control). If left untreated, these STIs cause significant health risks such as pelvic inflammatory disease (PID) in women, which can lead to infertility, chronic pelvic pain and ectopic pregnancy [1]. Bacterial vaginosis (BV) is the most common vaginal condition reported by women and evidence suggests that this condition can also be sexually transmitted [2, 3]. BV is associated with preterm birth, endometritis and increased risk of acquisition and transmission of STIs, including *C. trachomatis*, *N. gonorrhoeae*, and HIV [4–9]. While BV is a complex disorder thought to arise from the overgrowth of a wide array of anaerobes [10–12] and a decreased proportion of *Lactobacillus* spp. [13], *Gardnerella vaginalis* often predominates during BV and is thought to form a biofilm that is associated with BV recurrence [14, 15].

The constituents of the vaginal microbiota can affect susceptibility to STIs, though little is known about the molecular mechanisms at work in these interactions. In particular, women with *Lactobacillus*-dominated vaginal microbiota are at lower risk of contracting *N. gonorrhoeae* and other STIs [16–19]. *Lactobacillus* spp. inhibit growth of *N. gonorrhoeae in vitro*, and inhibitory strains are more prevalent in women not infected with gonorrhea [20]. Depending on growth conditions, *Lactobacillus* spp*.* inhibit *N. gonorrhoeae* and *G. vaginalis*, through production of hydrogen peroxide (H_2_O_2_), lactic acid and/or secreted proteins [17, 21–28]. It is unlikely though that H_2_O_2_ plays a major role *in vivo* as physiological concentrations are below that required for inhibition of BV-associated bacterial growth [26]. The majority of investigations into the interactions of *Lactobacillus* spp. with *N. gonorrhoeae* and *G. vaginalis* have been performed *in vitro*. While this work has provided valuable insights into these interactions, more complex models are required to expand our knowledge of how vaginal *Lactobacillus* spp. influence pathogen attachment, growth and virulence in the female reproductive tract.

While mouse models of both *N. gonorrhoeae* and *G. vaginalis* infection have been developed, these models are limited by the fact that the mouse vaginal epithelium in keratinized (unlike the squamous epithelium of the human vaginal epithelium), and that the resident mouse microbiota has been somewhat characterized and is not similar to that of humans [29]. Further, the mouse vaginal pH, which is thought to be a critical factor in STI protection, is higher than that of humans and it is unclear how well results in mice extrapolate to humans [30–33]. Mice are also relatively expensive and low throughput. Our limited understanding of vaginal microbial interactions with one another and with host mucosa highlight the need for a new model that will allow these questions to be addressed in a biologically complex and defined environment.

Porcine vaginal mucosa (PVM) may represent a novel model for STI interactions with the resident microbiota and the host mucosal surface. PVM is an excellent model of the human vagina. As in humans, PVM is composed of a stratified, squamous epithelium with a similar surface lipid composition and underlying resident immune infiltrate [34]. PVM is also similar to the human vagina in pH and permeability characteristics. An *ex vivo* PVM model has been used extensively to study the interactions of *Staphylococcus aureus* and its virulence factors with the host mucosa, as well as to investigate biofilm formation and antimicrobial efficacy [35–46]. The similarity of PVM to the human vaginal mucosa, and its demonstrated use as a model for pathogen/host interactions and biofilm formation make it an excellent candidate for similar investigations with STI pathogens.

In the current study, the *ex vivo* PVM tissue model was used to explore the role of cervicovaginal *Lactobacillus* spp. in affecting colonization and growth of *G. vaginalis* and *N. gonorrhoeae*. It was hypothesized that human clinical isolates of *Lactobacillus* spp., *G. vaginalis* and *N. gonorrhoeae* can colonize and grow on *ex vivo* PVM, form biofilm (*G. vaginalis* and *N. gonorrhoeae*), and that interactions of these organisms could be investigated using the PVM model. The data presented support these hypotheses and demonstrate a clear role for pH and *Lactobacillus* in inhibition of pathogen growth on live vaginal mucosa.

## Results

### Human clinical isolates grow and form biofilm on PVM

**Fig. 1:**
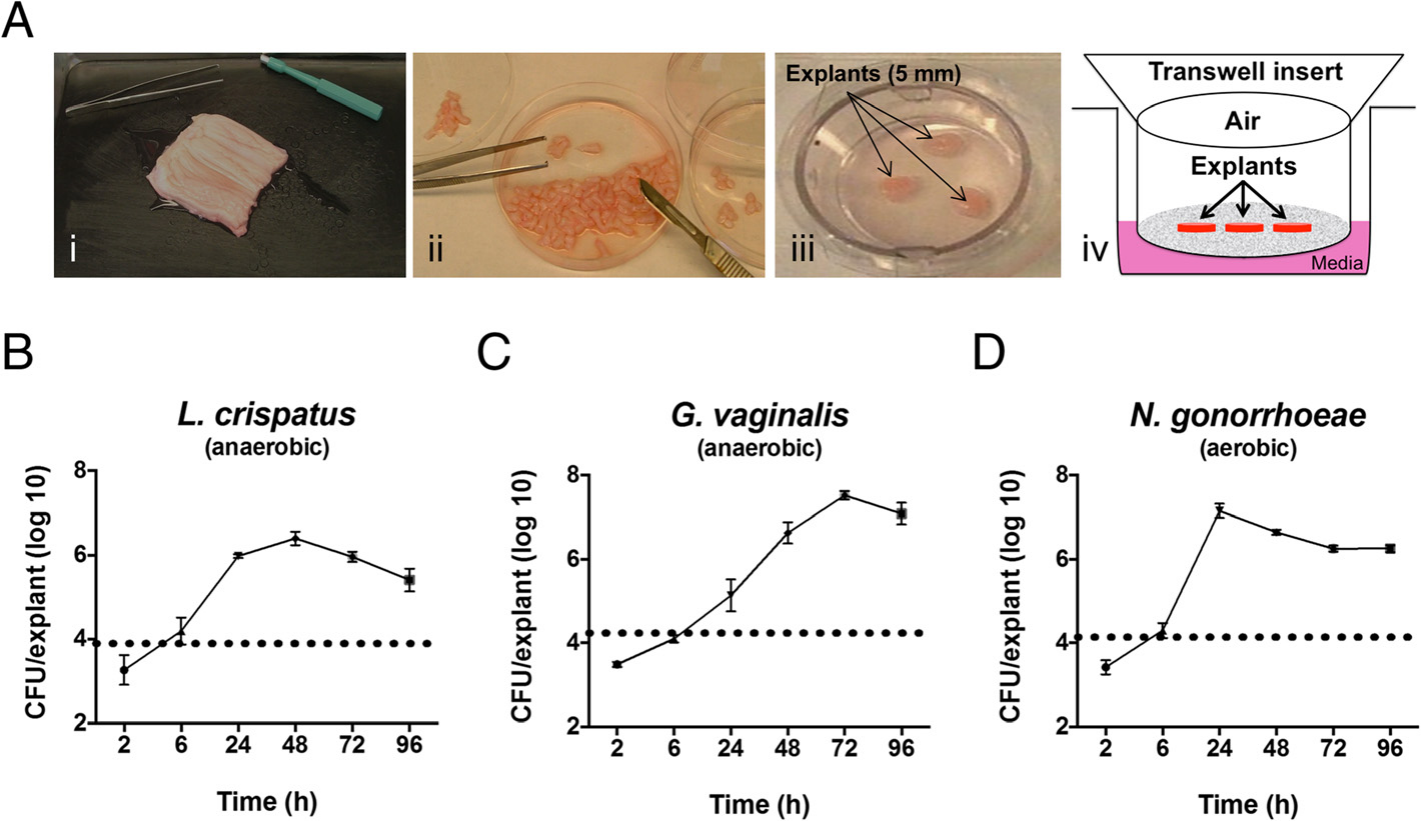
Growth of human clinical isolates on porcine vaginal mucosa (PVM). **a** PVM is obtained as large specimens (i) and 5 mm explants are trimmed (ii) and placed mucosal side up in transwells over liquid media (iii, iv). “Air” refers to either an aerobic or anaerobic environment depending on the experiment being performed. **b** *L. crispatus*, **c** *G. vaginalis*, and **d** *N. gonorrhoeae* were inoculated onto PVM explants at ~ 10^4^ CFU/explant (dotted lines). Explants were processed for CFU/explant at each time point to evaluate bacterial growth. Data was log_10_ transformed and plotted on a log scale as the mean ± SD

The PVM is obtained as relatively large specimens (~12 x 6 cm), from which 5 mm biopsies are taken and trimmed to produce mucosal explants (Fig. 1a) [37]. To determine if human clinical bacterial isolates can grow on PVM, explants were inoculated with ~10^4^ CFU/explant of *Lactobacillus* spp., *G. vaginalis*, and *N. gonorrhoeae. L. crispatus* consistently grew 2 to 3 logs, exhibiting peak growth at 48 h post-inoculation with ~ 2.6 x 10^6^ CFU/explant (~1.0 x 10^7^ CFU/ml) (Fig. 1b). *L. iners, L. jensenii,* and *L. gasseri* also showed maximal growth at 48 – 72 h with ~10^7^ CFU/explant, but they were not used for further experiments in the current study (data not shown). *G. vaginalis* grew to ~ 3.4 x 10^7^ CFU/explant (~1.3 x 10^8^ CFU/ml) with peak growth at 48 – 72 h (Fig. 1c). *N. gonorrhoeae* grew to ~ 1.1 x 10^7^ CFU/explant (~4.2 x 10^7^ CFU/ml) with peak growth at 24 – 48 h (Fig. 1d). It should be noted that *N. gonorrhoeae* grew best on the PVM when the underlying media was at pH 5.5 – 6.5 (see below). All *N. gonorrhoeae* experiments were performed under aerobic conditions, as *N. gonorrhoeae* did not grow on PVM anaerobically with RPMI as the underlying media. All other organisms were grown on PVM anaerobically.

**Fig. 2:**
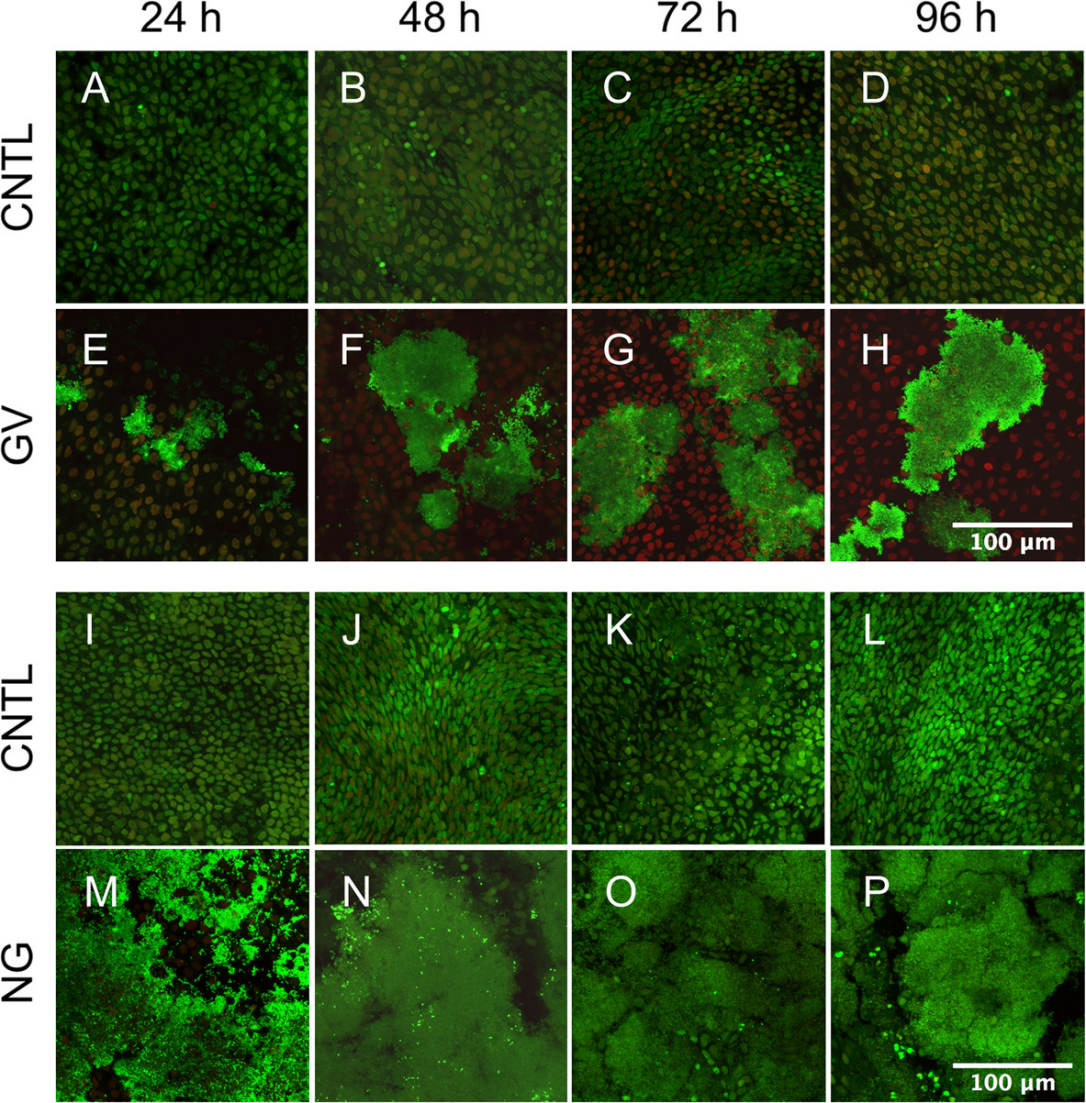
*G. vaginalis* and *N. gonorrhoeae* form biofilm on PVM. Strains were inoculated onto PVM explants at ~ 10^4^ CFU/explant and processed for microscopy at indicated times. The LIVE/DEAD stain allows for imaging of both the mucosal epithelium and bacteria. Green cells are alive while red cells are dead. **a**-**d**, **i**-**l** Uncolonized control (CNTL) tissue remains healthy throughout both experiments as evidenced by large green intact epithelial cells. **e**-**h** By 48 h post-colonization, *G. vaginalis* (GV) forms a patchy biofilm that persists and spreads over time (anaerobic growth). **m**-**p** In just 24 h *N. gonorrhoeae* (NG) forms a robust biofilm, which thickens and persists over time (aerobic growth). Epithelial cells that can be seen under and around the NG biofilm are alive as evidenced by their green staining, while those on GV-colonized explants are dead (large red cells under the green biofilm). Scale bars = 100 μm for all images

*N. gonorrhoeae* can form a biofilm in continuous-flow chambers and on human cervical epithelial cells, suggesting that biofilm formation *in vivo* may play a role in pathogenesis [47, 48]. Similarly, *G. vaginalis* can form a biofilm *in vitro* and *in vivo* that might contribute to the resistance of BV to standard treatments [49–52]. The ability of *N. gonorrhoeae* and *G. vaginalis* to form biofilm on PVM was assessed in order to determine if the PVM model can be used to study the mechanisms of biofilm formation. A LIVE/DEAD stain was used with confocal microscopy to visualize both biofilm development and the health of PVM epithelium. By 24 h post-inoculation, single and microcolonies of adherent bacteria were observed on PVM explants colonized with *G. vaginalis* (Fig. 2e). Where these colonies were observed, the underlying epithelium showed a mix of live (green) and dead (red) cells, whereas on areas of tissue that did not show bacterial colonization, the epithelium appeared mostly green as in the uninfected controls (Fig. 2a). By 48 h, patchy *G. vaginalis* biofilm was observed that thickened and colonized more of the surface area of the tissue over time (Fig. 2f-h). For these large areas of biofilm the underlying epithelium was either completely degraded or composed of dead cells (red). PVM colonized with *N. gonorrhoeae* exhibited robust biofilm development within 24 h (Fig. 2m) when compared to uninfected controls (Fig. 2i). By 48 h post-inoculation, *N. gonorrhoeae* formed a thick biofilm covering most of the surface of the explants (Fig. 2n-p). Control tissue remained healthy and uninfected over the course of the experiment (Fig. 2i-l). Unlike the epithelial toxicity observed with *G. vaginalis* biofilm, epithelial cells that could be seen between patches of *N. gonorrhoeae* biofilm were mostly green indicating that they were alive. Biofilm was not observed on explants colonized with *L. crispatus* alone at any time-point (data not shown).

### Low pH inhibits growth of *G. vaginalis* and *N. gonorrhoeae* on PVM

**Fig. 3:**
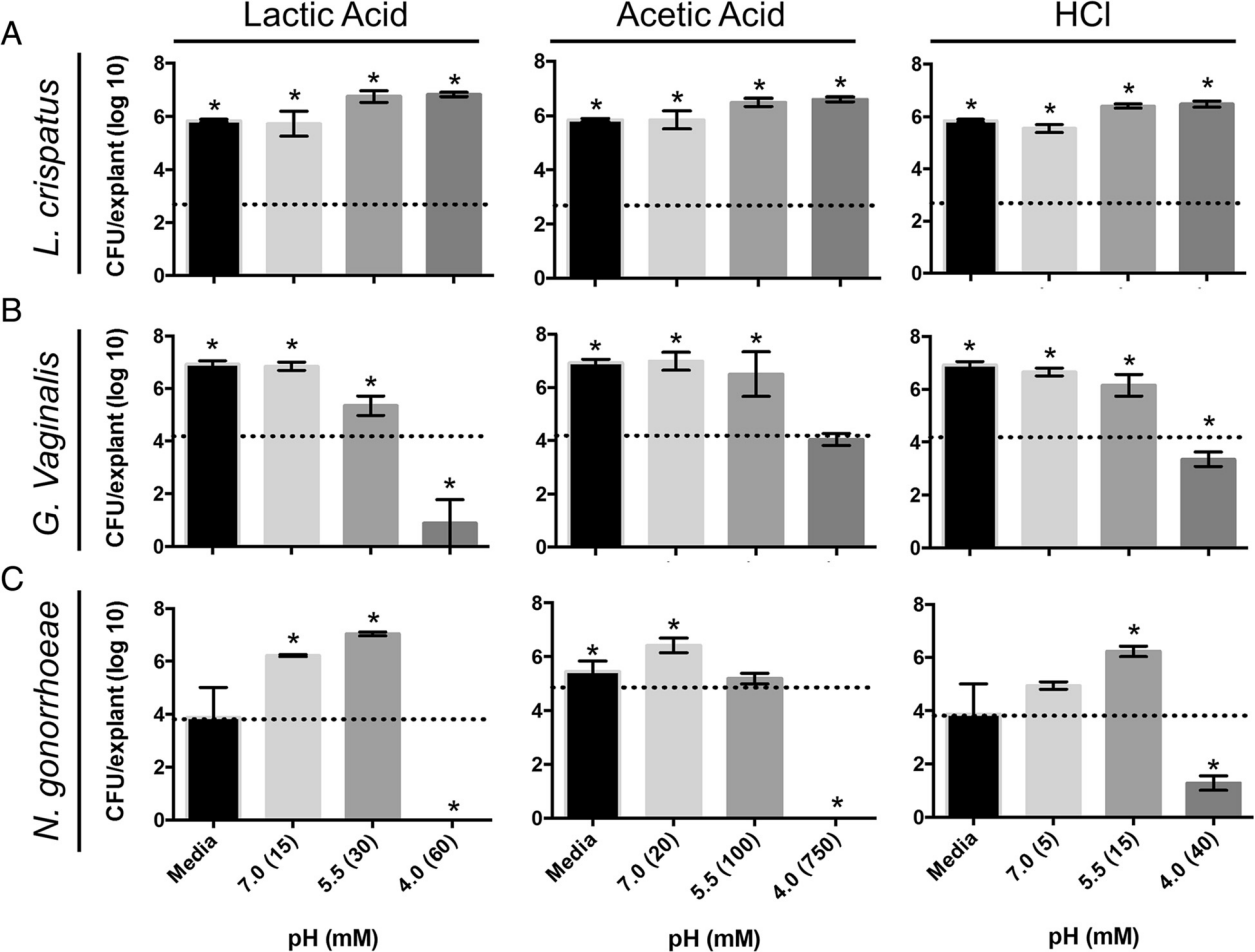
Various acids inhibit growth of *G. vaginalis* and *N. gonorrhoeae* on PVM. RPMI was pH-adjusted with lactic acid, acetic acid or hydrochloric acid (HCl) and placed under transwells containing PVM explants. Bacteria were enumerated 48 h post-colonization. *L. crispatus* and *G. vaginalis* were incubated anaerobically, while *N. gonorrhoeae* was incubated aerobically. **a** The low pH produced by each of the three acids has no effect on growth of *L. crispatus*. **b** *G. vaginalis* growth is inhibited at pH 4.0 in the presence of all three acids. **c** *N. gonorrhoeae* growth peaked at pH 5.5 in the presence of lactic acid and HCl, while growth was inhibited at pH 5.5 in the presence of acetic acid. Asterisks indicate significant difference from inoculum (dotted lines) (*p* < 0.0001)

Lactic acid is thought to play a critical and complex role in maintaining a healthy vaginal environment [23]. It is thought that one important function of lactic acid is inhibiting the growth of potentially pathogenic organisms including *G. vaginalis* and *N. gonorrhoeae*. Acetic acid is produced by *G. vaginalis* and other anaerobes and high levels of acetic acid in vaginal fluid are associated with BV [53–56]. To determine if lactic acid, acetic acid or acidity alone (using HCl) affect growth of the organisms under study on the PVM model, culture media containing acids at pH 7.0, 5.5 or 4.0 were produced and placed under transwell membranes containing explants prior to inoculation with various organisms. A reduction in pH with any of the three acids tested had no affect on growth of *L. crispatus* at 48 h post-inoculation (Fig. 3a). In contrast, *G. vaginalis* and *N. gonorrhoeae* were unable to grow on PVM in the presence of media adjusted to pH 4.0 with all three acids tested (Fig. 3b, c). Interestingly, while *N. gonorrhoeae* grew well over both lactic acid and HCl at pH 5.5, it consistently grew poorly over acetic acid at pH 5.5, regardless of inoculum.

### *L. crispatus* produces lactic acid and inhibits growth of *G. vaginalis *and *N. gonorrhoeae* on PVM

**Fig. 4:**
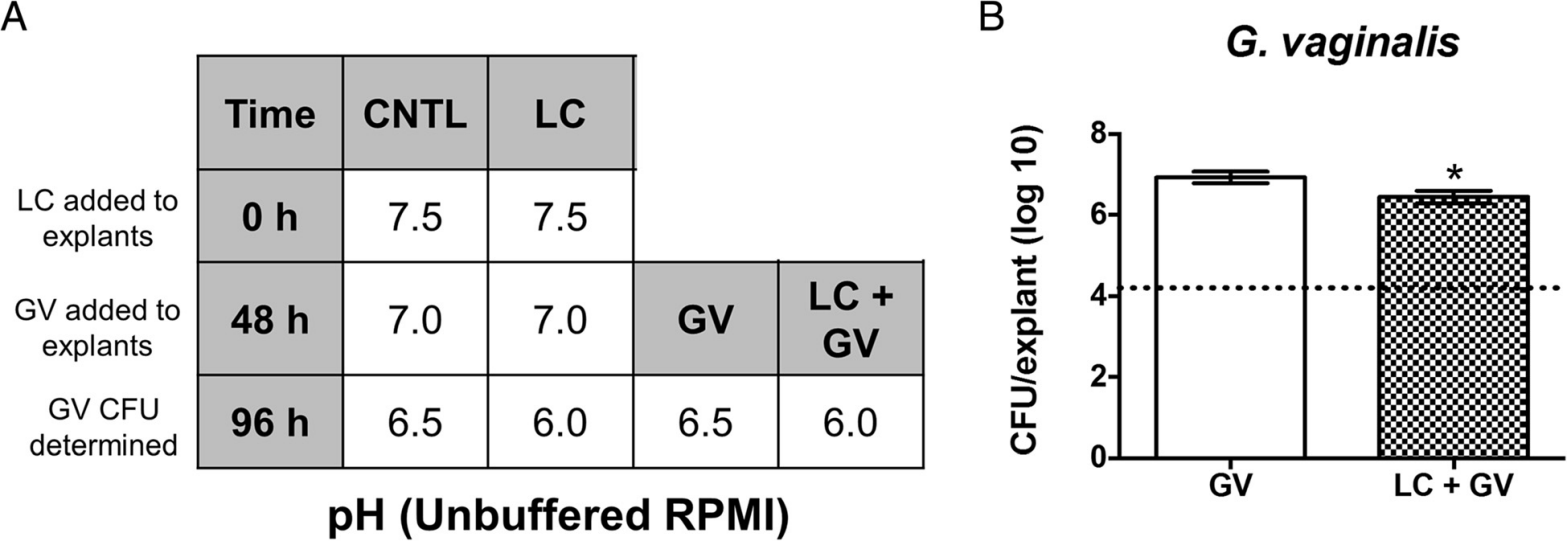
Unbuffered RPMI does not support sufficient pH changes in the presence of *L. crispatus* on PVM. Unbuffered RPMI was placed under transwells and relevant explants were inoculated with *L. crispatus*. After 48 h, relevant explants were inoculated with *G. vaginalis*. Experiments were performed under anaerobic conditions and the pH of media below explants was monitored over time. **a** Media under uncolonized controls (CNTL) and those colonized with *G. vaginalis* (GV) alone reached pH 6.5 while those colonized with *L. crispatus* (LC) alone or co-colonized with both organisms (LC + GV) reached pH 6.0 (*N* = 4, *n* = 1). **b** The presence of *L. crispatus* had a statistically significant (asterisk, *p* < 0.016), yet minor effect on *G. vaginalis* growth. Dotted line represents GV inoculum

*Lactobacillus* spp*.* inhibit *in vitro* growth of *G. vaginalis* and *N. gonorrhoeae* [20, 27, 57]. Co-colonization experiments were performed to determine if *L. crispatus* affects *G. vaginalis* and *N. gonorrhoeae* growth on PVM. Because inhibition of growth of *G. vaginalis* and *N. gonorrhoeae* is thought to rely in part on lactic acid production by vaginal *Lactobacillus* spp., resulting in low pH, unbuffered RPMI was initially used to maintain explants. PVM was inoculated with *L. crispatus* for 48 h prior to addition of *G. vaginalis* (all incubations were performed under anaerobic conditions). Under these conditions, the pH of the media under the transwells containing explants inoculated with *L. crispatus* did not go below 6.0 (Fig. 4a) and growth of *G. vaginalis* was only reduced by ~ ½ log (Fig. 4b).

It seemed likely that the relatively small number of bacteria on each explant was insufficient to produce enough lactic acid to lower the pH of the 1 ml of media below the transwells to < 6.0. It was hypothesized that use of media previously “conditioned” by *L. crispatus* would support meaningful pH changes in the presence of *L. crispatus*. To test this hypothesis, *L. crispatus* conditioned media (CM) produced by sterile filtering overnight aerobic cultures (produced without shaking) of *L. crispatus* (pH 4.0 – 4.5) was mixed with unbuffered RPMI resulting in a pH of 5.5 – 6.0, which is permissible for growth of *G. vaginalis* (Fig. 3). This allowed for testing the ability of *L. crispatus* on PVM to lower the local pH to that observed in the vagina of healthy human subjects (~ pH 3.5 – 4.5) and inhibit growth of *G. vaginalis*.

**Fig. 5:**
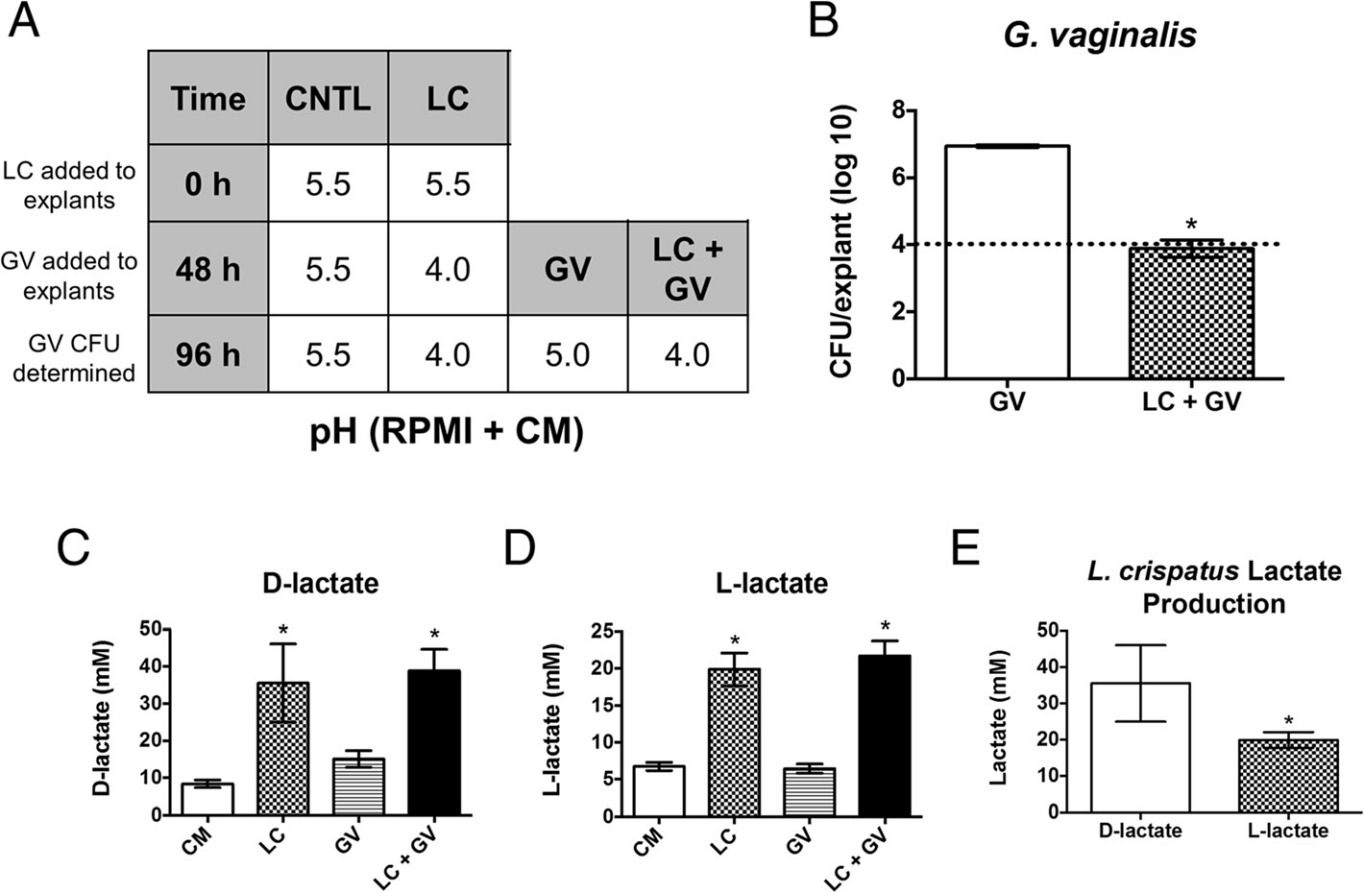
Conditioned media supports *L. crispatus*-induced pH changes, inhibiting growth of *G. vaginalis* on PVM. Media from overnight aerobic broth culture of *L. crispatus* was sterile filtered and mixed with unbuffered RPMI to achieve pH 5.5. This conditioned media (CM) was placed under transwells and relevant explants were inoculated with *L. crispatus*. After 48 h, relevant explants were inoculated with *G. vaginalis*. Experiments were performed under anaerobic conditions and the pH of media below explants was monitored over time. **a** Media under uncolonized controls (CNTL) and those colonized with *G. vaginalis* (GV) alone remained at pH 5.0 – 5.5 while those colonized with *L. crispatus* (LC) alone or co-colonized with both organisms (LC + GV) were reduced to pH 4.0 (*N* = 6, *n* = 1). **b** The presence of *L. crispatus* inhibited *G. vaginalis* growth. Dotted line represents GV inoculum. Asterisk indicates significant difference in growth between the two groups shown (*p* < 0.0001). **c**, **d** D- and L-lactate levels in the CM below transwells from experiment (**a**) were analyzed at 96 h. CM below explants colonized with *L. crispatus* (LC and LC + GV) showed a significant increase in lactic acid when compared with uncolonized controls (CM) and *G. vaginalis* (GV) alone (*p* < 0.0001). **e** A comparison of D- and L- lactate levels in CM below LC alone infections shows that D-lactate is produced at ~2X the level of L-lactate (*p* < 0.002). (C-E) Data shown are combined (*N* = 5, *n* = 1)

After 48 h, *L. crispatus* lowered the pH of CM below the transwells to 4.0, a pH that was maintained over the course of the experiments (Fig. 5a). The CM below transwells containing uninfected explants or those colonized with *G. vaginalis* alone remained at pH 5.0 – 5.5. When *G. vaginalis* was added to explants pre-inoculated with *L. crispatus*, it failed to grow whereas *G. vaginalis* placed on explants alone (over CM, pH 5.5) grew to normal densities (Fig. 5b). The levels of D- and L-lactic acid were significantly increased in CM under transwells containing explants inoculated with *L. crispatus* but not those that were uninfected or colonized with *G. vaginalis* alone (Fig. 5c, d). D-lactate levels produced by *L. crispatus* alone averaged 35.52 ± 10.55 mM and L-lactic acid levels averaged 19.90 ± 2.22 mM over 5 experiments (Fig. 5e). These data are in excellent agreement with results showing that 60 mM lactic acid (D + L) was required to lower the pH to 4.0 and inhibit growth of *G. vaginalis* (Fig. 3b). It should be noted that the levels of D- and L-lactic acid reported in the “CM” columns of Fig. 5c and d are from overnight aerobic broth culture (obtained without shaking). Further accumulation of D- and L-lactic acid in this media (reported in Fig. 5c-e) was achieved under anaerobic conditions on PVM.

**Fig. 6:**
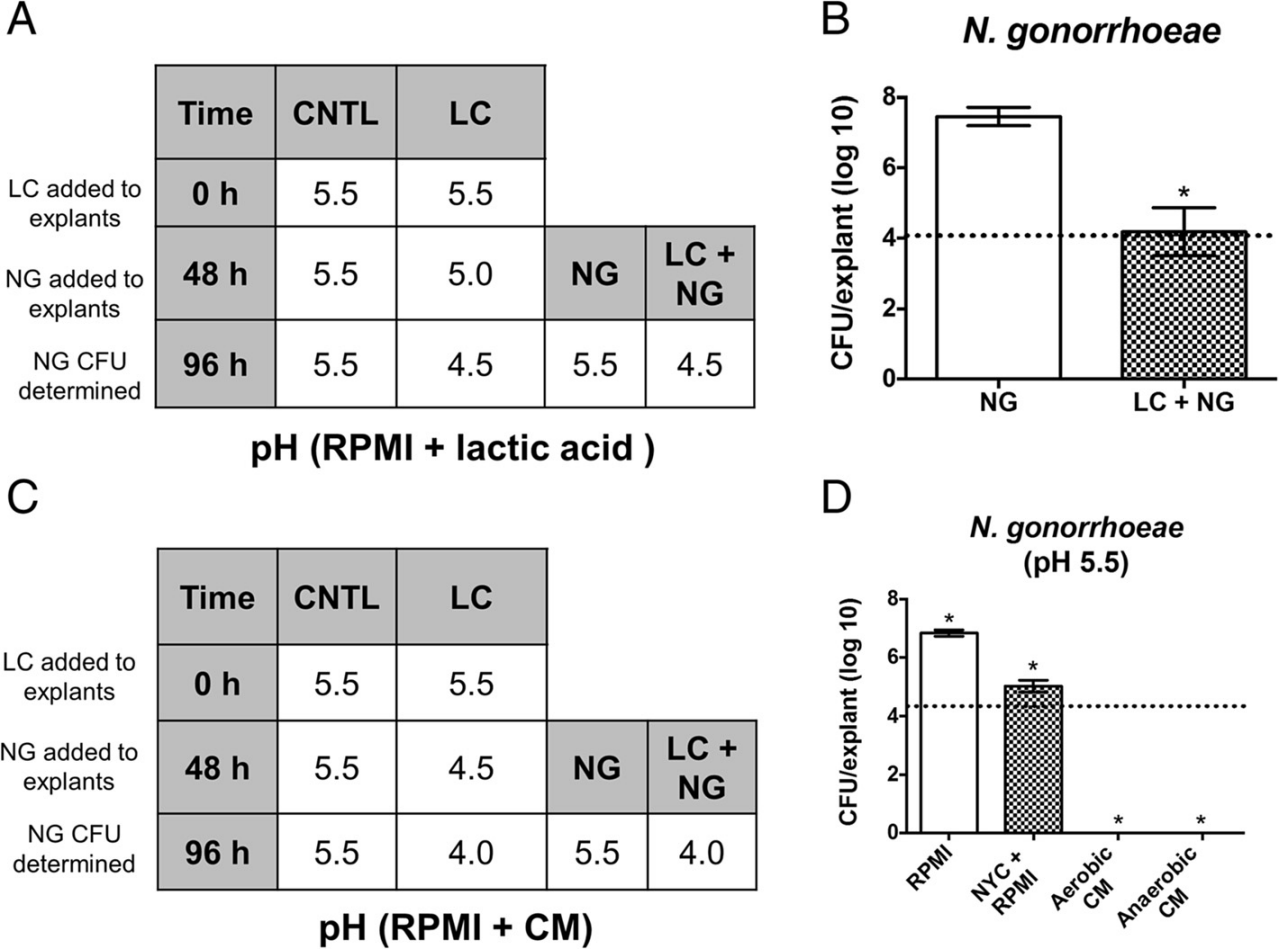
*L. crispatus* inhibits growth of *N. gonorrhoeae*. **a**, **b** Unbuffered RPMI or (**c**) *L. crispatus* CM was adjusted to pH 5.5 and placed under transwells. Relevant explants were inoculated with *L. crispatus*. After 48 h, relevant explants were inoculated with *N. gonorrhoeae*. Experiments were performed under aerobic conditions and the pH of media below explants was monitored over time. **a** RPMI under uncolonized controls (CNTL) and those colonized with *N. gonorrhoeae* (NG) alone remained at pH 5.5 while RPMI under explants colonized with *L. crispatus* (LC) alone or co-colonized with both organisms (LC + NG) was reduced to pH 4.5 (*N* = 4, *n* = 1). **b** The presence of *L. crispatus* inhibited 48 h *N. gonorrhoeae* growth. Asterisk indicates significant difference in growth between the two groups shown (*p* < 0.002). **c** CM under CNTL or NG remains at pH 5.5 while CM under explants colonized with LC alone or co-colonized with both organisms (LC + NG) was reduced to pH 4.0 (*N* = 3, *n* = 1). **d** While *N. gonorrhoeae* did exhibit a growth defect over NYC + RPMI media (the background media for CM) it was completely killed by aerobic or anaerobic CM at pH 5.5, even in the absence of a *L. crispatus* co-infection. Asterisks indicate significant difference from inoculum (*p* < 0.0001). For (**b**) and (**d**), dotted lines represent NG inocula

To directly analyze the effect of *L. crispatus* on *N. gonorrhoeae* growth, experiments were performed with unbuffered RPMI that was adjusted to pH 5.5 with lactic acid prior to inoculation. After 48 h, *L. crispatus* lowered the pH of media below transwells to 5.0 and further lowered the pH to 4.5 by 96 h (Fig. 6a). When *N. gonorrhoeae* was added to explants pre-inoculated with *L. crispatus*, the organism failed to grow whereas *N. gonorrhoeae* placed on explants alone grew to normal levels (Fig. 6b). The effect of *L. crispatus* CM on *N. gonorrhoeae* growth was also determined. After 48 h *L. crispatus* lowered the pH of underlying CM to 4.5 and further to pH 4.0 after 96 h (Fig. 6c). The CM below transwells containing uninfected explants or those colonized with *N. gonorrhoeae* alone remained at pH 5.5. Surprisingly, *N. gonorrhoeae* did not survive over CM, even at an otherwise permissible growth pH (5.5) and in the absence of *L. crispatus* pre-inoculation (CFUs were zero for *N. gonorrhoeae* alone over CM or with *L. crispatus* over CM). The inability of *N. gonorrhoeae* to survive on PVM in the presence of CM was observed with CM produced from anaerobic and aerobic *L. crispatus* cultures obtained without shaking (Fig. 6d). Though the background media for CM (NYC + RPMI) did affect *N. gonorrhoeae* growth on PVM, it was not bactericidal as was observed with CM. These data indicate that *L. crispatus* 24-9-7 produces a factor in broth culture that kills *N. gonorrhoeae* even when the pH is permissible for *N. gonorrhoeae* growth.

## Discussion

Our limited understanding of the interactions of microbes and the importance of microbial structures such as biofilms in affecting the health of the human female reproductive system requires the development of predictive, biologically complex models. The goal of the current study was to use *ex vivo* PVM to model interactions of *Lactobacillus* with *G. vaginalis* and *N. gonorrhoeae. Lactobacillus* spp., and *G. vaginalis* colonize the human vagina, while *N. gonorrhoeae* is an obligate human pathogen that preferentially infects both the endo- and ectocervix [58–61]. The PVM is squamous epithelium similar to human vaginal and ectocervical epithelia making it an ideal candidate for *ex vivo* studies of these organisms. Human clinical isolates of *Lactobacillus* spp., *G. vaginalis*, and *N. gonorrhoeae*, colonized and grew on PVM to clinically relevant densities. *Lactobacillus* spp. grew to ~ 1.0 x 10^7^ CFU/ml, which is in accordance with published findings of 10^3^ – 10^9^ CFU/ml in vaginal specimens [62, 63]. Growth of *G. vaginalis* on PVM (~1.3 x 10^8^ CFU/ml) mimics the densities found during BV, as women without BV are colonized by < 2 x 10^7^ CFU/ml, while those with BV are colonized by > 2 x 10^7^ CFU/ml [64]. *N. gonorrhoeae* grew to ~ 4.2 x 10^7^ CFU/ml, which is slightly higher than the reported range of 5 x 10^3^ – 8 x 10^6^ CFU/ml obtained from vaginal washes [65]. These data demonstrate that the observed growth of human clinical bacterial isolates on PVM reflects the number of organisms found in human vaginal fluids/washes. Investigations of temporal dynamics on PVM are limited though as growth of the organisms under study generally peaked at 48 – 72 h post-colonization and waned at later time points, likely due to depletion of nutrients in this closed system.

Both *G. vaginalis* and *N. gonorrhoeae* form robust biofilms on PVM. Multiple lines of evidence suggest that biofilm formation by *G. vaginalis* strains *in vivo* is a critical first step in the development and recurrence of BV [66, 67]. Additionally, evidence suggests that the ability of any particular strain to form a biofilm may be a key factor in distinguishing pathogenic *G. vaginalis* from commensal strains found in many asymptomatic women [68]. The *G. vaginalis* strain used in this study (ATCC 14018) produces a cholesterol-dependent cytolysin (CDC), vaginolysin (VLY) [69, 70] that lyses cervical epithelial cells [71]. PVM colonized with *G. vaginalis* biofilm showed extensive epithelial cell death, suggesting that VLY might be active in this system. The *Staphylococcus aureus* cytolysin α-toxin is required for biofilm formation on PVM [36] while the *Streptococcus pneumoniae* CDC, pneumolysin, is not required for biofilm formation in a mouse nasopharyngeal colonization model [72]. It will be interesting to determine if VLY is required for biofilm formation *ex vivo* and what role lysis versus epithelial cell signaling may play in the VLY contribution to *G. vaginalis* colonization and/or biofilm formation [69]. Because *G. vaginalis* is a genomically diverse species, other factors might be important in determining characteristics necessary for pathogenicity [73]. Future experiments will explore the molecular mechanisms used by *G. vaginalis*, and *N. gonorrhoeae* to form biofilm on PVM and the ability of *Lactobacillus* spp. and their secreted products to prevent/disrupt these biofilms.

Vaginal pH increases in women with BV and may be associated with STI susceptibility. It is becoming evident that lactic acid produced by *Lactobacillus* spp. contributes to overall vaginal health and inhibition of growth of pathogenic organisms *in vitro* [21, 23, 74]. BV-associated organisms, including *G. vaginalis,* produce acetic acid and there is a loss of lactic acid production and an increase in acetic acid in the vaginal fluid of women with BV [53, 56]. Of the three acids tested, lactic acid consistently showed a much larger degree of killing *G. vaginalis* at pH 4.0. It may be that lactic acid has a specific bactericidal affect on *G. vaginalis* but it is unclear if this affect is necessary to keep *G. vaginalis* growth in check, or if the effects of low pH alone are sufficient to do so.

Co-colonization experiments with *L. crispatus* and *G. vaginalis* on PVM (anaerobic) clearly showed an association between lactic acid production, a reduction in the local pH, and inhibition of *G. vaginalis* growth. These results reflect previous observations that under anaerobic conditions (where H_2_O_2_ is minimally produced), lactic acid inhibits growth of *G. vaginalis* (and other BV-associated organisms) *in vitro* [26]. The results here show that *L. crispatus* inhibition of *G. vaginalis* growth is pH-dependent; lactic acid production is not sufficient to inhibit *G. vaginalis* growth if it is not accompanied by a reduction in pH. *L. crispatus* produced higher levels of D- versus L-lactate, as previously reported for the species [75]. The differential production of D- and L-lactate by various *Lactobacillus* spp. may be important in influencing host responses and susceptibility to BV and future work will tease out these differences by expanding the species and strains used in co-infection experiments.

Women with *Lactobacillus*-dominated vaginal microbiota are less susceptible to *N. gonorrhoeae* infection [16, 19, 20]. The mechanism of inhibition of *N. gonorrhoeae* by *Lactobacillus* spp. is unclear as multiple studies demonstrate that lactic acid, H_2_O_2_, and *Lactobacillus* bacteriocins can inhibit *N. gonorrhoeae* growth *in vitro* depending on the growth conditions. Though lactic acid alone at pH 5.5 was permissible for *N. gonorrhoeae* growth, *L. crispatus* anaerobic CM at pH 5.5 was not, indicating that this *L. crispatus* strain produces a factor other than lactic acid that inhibits *N. gonorrhoeae* growth. This data also supports the hypothesis that H_2_O_2_ does not likely play an inhibitory role *in vivo,* as H_2_O_2_ is undetectable in *Lactobacillus* broth culture incubated anaerobically and in cervicovaginal fluid [25, 76].

PVM represents a biologically relevant model that cannot only be used to ask fundamental questions about microbial interactions, but also help translate new technologies to clinical use. While it is likely that some host/bacterial interactions are not identical between the porcine and human mucosal epithelia, PVM has proven to be useful in assessing host toxicity of potential antimicrobials and is predictive of clinical efficacy [38, 77]. The use of probiotic *Lactobacillus* spp. has been investigated as a prevention of and treatment for BV [78]. The PVM model used in this study could be used to screen potential probiotic organisms for their ability to prevent the growth of a variety of BV-associated organisms and decipher the mechanisms of inhibition. Advantages of PVM include that it is not subject to regulation as live animal models are, it is inexpensive, relatively easy to obtain and semi-high throughput. This model is potentially an excellent platform for initial testing of novel therapeutics against STIs, such as drug-resistant strains of *N. gonorrhoeae*. Importantly, toxic effects of new drugs on the host mucosa and resident protective microbes such as *Lactobacillus* spp. could also be investigated with the PVM model.

Future work will include investigations of the mechanisms of biofilm formation, *Lactobacillus* spp. interactions with BV-associated organisms and STI agents, the host response to colonization and the efficacy of antimicrobials against STIs using the PVM model.

## Conclusions

An acidic environment (pH <4.5) inhibits colonization of live vaginal mucosa by both *G. vaginalis* and *N. gonorrhoeae* regardless of the acid tested. *N. gonorrhoeae* grows best in the presence of lactic acid at pH 5.5 perhaps contributing to increased susceptibility during BV. Additionally, a high concentration of acetic acid inhibits *N. gonorrhoeae* growth on PVM at pH 5.5. A stable *L. crispatus* colonization of live vaginal mucosa is able to prevent colonization of *G. vaginalis* in a pH-dependent manner, while *L. crispatus* secretes a factor that kills *N. gonorrhoeae*. The PVM model will continue to be used to investigate these interactions and the mechanisms of *G. vaginalis* and *N. gonorrhoeae* biofilm formation on live vaginal tissue.

## Methods

### Bacterial isolates and culture conditions

*L. crispatus, L. jensenii, L. gasseri* and *L. iners* were isolated from vaginal swabs of reproductive age, asymptomatic women who were not menstruating at the time of collection. These isolates were collected as part of broader longitudinal genomic studies of the vaginal microbiome [79, 80]. The clinical study protocols were approved by the Institutional Review Boards of the Johns Hopkins University School of Medicine, the University of Maryland School of Medicine and the University of Alabama at Birmingham. Written informed consent was obtained from all participants. The *L. crispatus* isolate used in the current study was designated 24-9-7 and was isolated from a woman who was dominated with *L. crispatus* each day of the 10-week sampling period except during menses when the community shifted and *L. iners* was dominant. *G. vaginalis* is a clinical isolate obtained from ATCC (14018). *G. vaginalis* and *Lactobacillus* spp. were cultured in New York City III (NYC III) media (10 g/L proteose peptone, 10 g/l beef extract, 5 g/l yeast extract, 5 g/L NaCl, 1.2 g/L MgSO_4_, 2 g/L MnSO_4_ ∙ H_2_O, 5.7 g/L K_2_HPO_4_, 20 g/L glucose, 10 % fetal bovine serum [FBS]) overnight anaerobically at 37 °C without shaking and diluted 1:10 or 1:100 just prior to inoculation. *N. gonorrhoeae* strain 23482 is a clinical isolate from male urethral source obtained from the Minnesota Department of Health. Bacteria from a freshly streaked modified Thayer-martin (MTM) agar plate (VWR, 90006–270) were scrapped into 1 ml of media followed by vortexing and diluting 1:100 for immediate use as *N. gonorrhoeae* inocula. Anaerobic conditions (<1.0 % O_2_, > 13 % CO_2_) for relevant experiments were achieved using the GasPak system (BD Biosciences, 260672, 260001). A spiral plater (Biotek, Microbiology International) was used for enumeration of CFUs. *Lactobacillus* spp. and *G. vaginalis* were plated on tryptic soy agar containing 5 % sheep’s blood (Fisher, B11947) and *N. gonorrhoeae* was plated on MTM agar.

### Ex vivo porcine vaginal mucosa

Specimens of normal porcine vaginal mucosa (PVM) are excised from mature (6 months), animals at slaughter in the University of Minnesota Andrew Boss Laboratory of Meat Science and transported to the laboratory in antibiotic-free RPMI 1640 (Gibco) with 10 % fetal calf serum. The vaginal tissue is a by-product of the slaughter of animals for human consumption and therefore is Institutional Animal Care and Use Committee (IACUC) exempt. Tissue was utilized within 3 h of excision. Explants of uniform size were obtained using a 5 mm biopsy punch and excess muscle was trimmed away with a scalpel. Explants were sterilized via 1 min incubation with 10 % povidone-iodine (PI) (Alfa Aesar, 45782). The explants were rinsed once with 10 ml standard sampling solution (3 mM KH_2_PO_4_, 71 mM Na_2_HPO_4_, 0.1 % Triton X-100, 3 % Tween 80, 3 g/L lecithin, 4 mM Na_2_S_2_O_3_ ∙ 5H_2_O) to neutralize PI and three times with 10 ml RPMI and placed mucosal side up on a PET track-etched 0.4 mm cell culture insert (Fisher, 0877115) in 6-well plates containing 1 ml of indicated media below inserts. The mucosal surface was continually exposed to the aerobic or anaerobic environment.

For bacterial colonization of PVM, explants were inoculated with ~10^4^ CFU/explant. *Lactobacillus* spp. and *G. vaginalis* were incubated anaerobically while *N. gonorrhoeae* was incubated aerobically on explants for indicated times at 37 °C. Explants were vortexed in 250 μl PBS for 4 min at max speed to release bacteria for analysis of CFU/explant and CFU/ml.

To determine the effects of various acids on bacterial growth: lactic acid (Sigma, 69785) was used at 15 mM (pH 7.0), 30 mM (pH 5.5) or 60 mM (pH 4.0); acetic acid was used at 20 mM (pH 7.0), 100 mM (pH 5.5) or 750 mM (pH 4.0); hydrochloric acid (HCl) was used at 5 mM (pH 7.0), 15 mM pH 5.5) or 40 mM (pH 4.0). To achieve RPMI at pH 7.0 for each acid, the acids were added to RPMI at the indicated molarity and the solution pH-adjusted with NaOH. Explants were inoculated with ~10^4^ CFU/explant and incubated for 48 h at 37 °C over indicated media. *L. crispatus* and *G. vaginalis* were incubated anaerobically while *N. gonorrhoeae* were incubated aerobically. Explants were then processed as above.

For co-colonization experiments, overnight cultures of *L. crispatus* (pH 4.0) were sterile filtered (0.45 μm) to produce conditioned media (CM). CM was diluted by ~ ½ with unbuffered RPMI 1640 (GIBCO, 11875–093) to reach pH 6.0 – 5.5. As indicated in figures, either RPMI + CM, RPMI + lactic acid or unbuffered RPMI was used for each experiment. Explants were washed for 30 min in relevant media and placed in transwells over 1 ml of corresponding media. Explants were inoculated with ~10^4^ *L. crispatus* CFU/explant and incubated anaerobically for 48 h at 37 °C. Explants were then inoculated with ~10^4^ *G. vaginalis* or *N. gonorrhoeae* CFU/explant and incubated anaerobically or aerobically, respectively, for 48 h. Explants were processed as above for CFU analysis. The pH of the underlying media was recorded at 0 h, 48 h and 96 h.

### Analysis of lactic acid production

The media below transwells used in the *L. crispatus/G. vaginalis* co-colonization experiments described above were collected at the end of the experiment (96 h) and stored at 4 °C for further analysis. Levels of D- and L- lactic acid in the media were assessed using a lactate quantification assay kit according to the manufacturer’s instructions (BioAssay Systems, EFDLC-100 and EFLLC-100).

### Biofilm microscopy

Uninfected and PVM explants colonized with various bacterial strains and incubated for 24 – 96 h at 37 °C were stained using the LIVE/DEAD Biofilm Viability kit (Invitrogen, L10316) according to instructions. Explants were washed three times with 1 ml/well in Hank’s balanced salt solution (Life Technologies, 14185052) and transferred to glass slides. A 1 mm spacer (Electron Microscopy Sciences, 70327–10) with a glass coverslip was placed over the explants. The epithelial surface of the explants was imaged with a Nikon Ni-E confocal microscope using a 60X oil immersion objective. Images were captured and processed using the Nikon NIS Elements software. Imaging was performed at the University Imaging Centers at the University of Minnesota.

### Statistical analysis

All data shown are representative of at least 3 independent experiments with at least 3 replicates (*N* ≥ 3, *n* ≥ 3) unless otherwise noted. Graphs were produced and statistical analysis performed using the Prism software (GraphPad). Statistical differences were determined using either One-Way ANOVA (with Dunnett’s multiple comparison test) or (when only two conditions were being compared) Student’s *t*-test.

## Availability of supporting data

The data set(s) supporting the results of this article is (are) included within the article (and its additional file(s)).

## Abbreviations

BV: Bacterial vaginosis
STI: Sexually transmitted infection
PVM: Porcine vaginal mucosa
PID: Pelvic inflammatory disease
H_2_O_2_: Hydrogen peroxide
HCl: Hydrochloric acid
CM: Conditioned media
CDC: Cholesterol dependent cytolysin
VLY: Vaginolysin
